# Supplementary material for: Attitude of youth towards self-employment: Evidence from university students in Yemen
Source: PLoS One. 2021 Sep 13;16(9):e0257358. doi: 10.1371/journal.pone.0257358 (PMC8437303; doi:10.1371/journal.pone.0257358)
Supplement: S1 Appendix — Code, constructs and items. (DOCX) [file pone.0257358.s001.docx]

S1 Appendix.

**Self-Employment Intention Questionnaire (SEIQ)**. code, construct and items.

| No | Code | Construct and Item | Score |
| --- | --- | --- | --- |
|  | **SEI** | **Self-employment Intention** | **1-5** |
| 1 | SEI1 | I am ready to do anything to become an entrepreneur/self-employed. | 1-5 |
| 2 | SEI2 | My career goal is to become an entrepreneur/self-employed. | 1-5 |
| 3 | SEI3 | I prefer to be self-employed rather than to be an employee in a big company. | 1-5 |
| 4 | SEI4 | I decided to set up a business in the future. | 1-5 |
|  | **ATE** | **Attitude Toward Entrepreneurship** | 1-5 |
|  | **ATEM** | **Attitude Toward Earn Money** | **1-5** |
| 5 | ATEM1 | To me, high income is an important criterion in assessing the level of personal success. | 1-5 |
| 6 | ATEM2 | It is important for me to make a lot of money. | 1-5 |
| 7 | ATEM3 | I firmly believe money can solve all my problems. | 1-5 |
| 8 | ATEM4 | I can only make a lot of money if I am an entrepreneur/self-employed. | 1-5 |
|  | **ATPS** | **Attitude Toward Personal Satisfaction as an entrepreneur** | **1-5** |
| 9 | ATPS1 | Being an entrepreneur/self-employed would give me great satisfaction. | 1-5 |
| 10 | ATPS2 | To me, becoming an entrepreneur/self-employed is to achieve a higher position from myself in society. | 1-5 |
| 11 | ATPS3 | To me, becoming an entrepreneur/self-employed is to be more respected by friends. | 1-5 |
|  | **ATQL** | **Attitude Toward Personal Quality of Life** | **1-5** |
| 12 | ATQL1 | Being an entrepreneur/self-employed implies improving my quality of life. | 1-5 |
| 13 | ATQL2 | Being an entrepreneur/self-employed implies greater flexibility for my personal and family life. | 1-5 |
| 14 | ATQL3 | Being an entrepreneur/self-employed implies contribute to the welfare of my relatives. | 1-5 |
| 15 | ATQL4 | Being an entrepreneur/self-employed implies increase the status and prestige of my family. | 1-5 |
|  | **ATDI** | **Attitude Toward Desire for Independence (Autonomy)** | **1-5** |
| 16 | ATDI1 | Independence at work is important for me*. | 1-5 |
| 17 | ATDI2 | I prefer to found my own business than to be an employee of a big company. | 1-5 |
| 18 | ATDI3 | I would rather be my own boss than have a secure job. | 1-5 |
| 19 | ATDI4 | I prefer to be an entrepreneur/self-employed to be able to choose my own work tasks. | 1-5 |
|  | **SNSV** | **Subjective Norms and Social Valuation** | **1-5** |
| 20 | SNSV1 | My family would approve of the decision to be an entrepreneur/self-employed. | 1-5 |
| 21 | SNSV2 | My close friends would approve of the decision to be an entrepreneur/self-employed. | 1-5 |
| 22 | SNSV3 | My classmates would approve of the decision to be an entrepreneur/self-employed. | 1-5 |
| 23 | SNSV4 | In my society, entrepreneurial activity is worthwhile, despite the risks. | 1-5 |
| 24 | SNSV5 | The culture in my society is highly favourable toward entrepreneurial activity. | 1-5 |
|  | **PBC** | **Perceived Behavioral Control** | **1-5** |
| 25 | PBC1 | Opening and operating a business are easy/not difficult for me. | 1-5 |
| 26 | PBC2 | I can control the creation process of a new business. | 1-5 |
| 27 | PBC3 | I would have complete control over the situation if I start and run a business. | 1-5 |
| 28 | PBC4 | I know all about the necessary practical details needed to start a business. | 1-5 |
| 29 | PBC5 | If wanted to, I could easily start and run a business. | 1-5 |
| 30 | PBC6 | If I tried to start a business, I would have a high probability of succeeding. | 1-5 |
| 31 | PBC7 | I know how to develop an entrepreneurial project. | 1-5 |
|  | **PBE** | **Perceived Barriers to Entrepreneurship** |  |
|  | **PBLC** | **Perceived Barriers Lack of capital** | **1-5** |
| 32 | PBLC1 | Difficulty in obtaining finance. | 1-5 |
| 33 | PBLC2 | Lack of own savings and assets. | 1-5 |
| 34 | PBLC3 | Lack of support from family or friends. | 1-5 |
| 35 | PBLC4 | Banks do not readily give credit to start-up companies. | 1-5 |
| 36 | PBLC5 | High-interest rates on loans from the financial institution. | 1-5 |
|  | **PBLK** | **Perceived Barriers Lack of Knowledge** | **1-5** |
| 37 | PBLK1 | Lack of marketing knowledge and skills. | 1-5 |
| 38 | PBLK2 | Lack of experience in management and accounting. | 1-5 |
| 39 | PBLK3 | Lack of information about business start-ups. | 1-5 |
| 40 | PBLK4 | Lack of business training. | 1-5 |
| 41 | PBLK5 | Lack of knowledge of the business world. | 1-5 |
|  | **PBCS** | **Perceived Barriers Hard Realty** | **1-5** |
| 42 | PBHR1 | Current economic/political conditions. | 1-5 |
| 43 | PBHR2 | The uncertainty of the future. | 1-5 |
| 44 | PBHR3 | There is a high risk of starting a new business. | 1-5 |
| 45 | PBHR4 | Bad economic indicators in general. | 1-5 |
|  | **PBSC** | **Perceived Barriers Lack of Self-confidence** | **1-5** |
| 46 | PBSC1 | Fear of failure. | 1-5 |
| 47 | PBSC2 | Lack of ideas regarding what business to start. | 1-5 |
| 48 | PBSC3 | Difficulty in convincing others about the idea. | 1-5 |
|  | **PBLS** | **Perceived Barriers Lack of Support Structure** | **1-5** |
| 49 | PBLS1 | Compliance with government regulations. | 1-5 |
| 50 | PBLS2 | High taxes and fees. | 1-5 |
| 51 | PBLS3 | Lack of formal help to start a business. | 1-5 |
| 52 | PBLS4 | Lack of organizations to assist entrepreneurs. | 1-5 |
| 53 | PBLS5 | The bureaucratic procedures for founding a new business. | 1-5 |
|  | **PSE** | **Perceived Support to Entrepreneurship** | **1-5** |
|  | **PES** | **Perceived Educational/University Support** | **1-5** |
| 54 | PES1 | The education in university improved my entrepreneurial spirit. | 1-5 |
| 55 | PES2 | My university provides the necessary knowledge about entrepreneurship. | 1-5 |
| 56 | PES3 | My university developed my entrepreneurial competence and skills. | 1-5 |
| 57 | PES4 | My university arranges conferences/workshops on entrepreneurship. | 1-5 |
|  | **PGS** | **Perceived Governmental Support** | **1-5** |
| 58 | PGS1 | The government shows a willingness to help individuals who want to be an entrepreneur. | 1-5 |
| 59 | PGS2 | The government provides necessary support to new businesses. | 1-5 |
| 60 | PGS3 | The government institutions assist individuals with starting their own businesses. | 1-5 |
| 61 | PGS4 | Banks and government grants have available funds for starting new businesses. | 1-5 |
|  | **PIS** | **Perceived Institutional Support** | **1-5** |
| 62 | PIS1 | In Yemen, entrepreneurs are encouraged by an institutional structural system including private, public, and non-governmental organizations. | 1-5 |
| 63 | PIS2 | Yemen’s economy provides many opportunities for entrepreneurs. | 1-5 |
| 64 | PIS3 | Taking loans from banks is easy/not difficult for entrepreneurs in Yemen. | 1-5 |
