# Supplementary material for: Attitude of youth towards self-employment: Evidence from university students in Yemen
Source: PLoS One. 2021 Sep 13;16(9):e0257358. doi: 10.1371/journal.pone.0257358 (PMC8437303; doi:10.1371/journal.pone.0257358)
Supplement: Attachment — Submitted filename: Response to Reviewers.docx [file pone.0257358.s003.docx]

**Response Letter to the Editor**

**Dear Dr.** **Camelia Delcea**

**Academic Editor**

**PLOS ONE**

We would like to thank you for consideration and correction suggestions by the reviewers and for the time & effort spent in reviewing our manuscript. The authors acknowledge and appreciate all comments given. We agree with the comments & thus we addressed them accordingly to the best of our knowledge. After the revision, we have written a point-by-point response letter to you as you can see below. Meanwhile, we also wrote a point-by-point response letter to the other two nice reviewers to acknowledge their help.

| **Manuscript Number:** | OPNE-D-21-17082 |
| --- | --- |
| **Manuscript Title:** | Attitude of youth towards self-employment: Evidence from university students in Yemen |

**Journal Requirements**:

**1) List of Reference**

Please review your reference list to ensure that it is complete and correct.

**Authors Response**

Thank you. We have revised the references list and we found that its complete & correct and complies with the requirements PLOS ONE references style. And according to the reviewer 1 comments, we added two references, they were highlighted with green color in the main body of the manuscript and the reference list. At the same time, we reduced the list of references (6 references) based on reviewer 1 comments.

**2) PLOS ONE’s Style Requirements**

Please ensure that your manuscript meets PLOS ONE’s style requirements, including those for file naming.

**Authors Response**

Thank you. We have ensured that our manuscript meets all journal’s style requirements, including journal’s template, files and figures naming, tables, etc.

**3) Participant Consent**

Please provide additional details regarding participant consent.

**Authors Response**

Thank you. Participants in our study were final-year undergraduate students, they have voluntarily completed the self-administered class questionnaire, Self-Employment Intention Questionnaire (SEIQ). First, the importance of participation in the research and the confidentiality of the data was emphasized, participants were informed that their consent is granted when they continue filling in the survey after reading the cover page, which emphasize the anonymity of study participants. Since data were gathered anonymously and analysed in aggregated form, we did not seek ethics committee approval as we do not expect study participants’ identities would be revealed nor harmed.

**4) Financial disclosure:**

Please clarify the sources of funding (financial or material support) for your study.

**Authors Response**

Thank you. Funding: The publication fee of this research was funded by Jilin University. The funder had no role in study design, data collection and analysis, decision to publish, or preparation of the manuscript.

**5) Supporting Information**

Please include captions for your Supporting Information files at the end of your manuscript.

**Authors Response**

Thank you. We have included captions of Supporting Information files at the end of the manuscript, and we have updated its citations on the body of the manuscript.

S1 Appendix. Self-Employment Intention Questionnaire

S1 Dataset. Data used in the study.

**Response Letter to the Reviewers**

| **SUMMARY OF COMMENTS AND CORRECTIONS** | | |
| --- | --- | --- |
| **No** | **REVIEWERS’ COMMENT** | **AUTHOR’S RESPONSE/ACTION** |
| **REVIEWER ONE** | | |
| 1 | The sentence in lines 43-44 "Currently, the rate of unemployment among higher-educated people remains proportionally more than the rate of less-educated workers" must be motivated by data with sources or references. | Thanks for the valuable comment. In the revised version, we have added two references that support our view, (Ayalew et al., 2018) and (Benda et al., 2019).  They were highlighted with green color in the main body of the test and the reference list. |
| 2 | Chapters must be enumerated to make the paper easier to follow. | Thank you. According to your comment, we have enumerated the study’s chapters. |
| 3 | For Chapter 2 (I guess) I would consider two separate bigger sub-section in this case: one for the behavioral factors and another one for barriers and environmental factors. In principle, the sub-section splitting should follow figure 1. | We appreciate your valuable suggestion. We believe that the current sub-sectioning is logical and tallies with study MAIN arguments. So, we have kept the original sectioning. |
| 4 | Why not merging the SEM subsection with the subsequent one? There are already too many sub-sections in the paper. | After considering your valuable suggestion, we merged some SEM subsections. Details are:   1) Under the “Structural Equation Modeling (SEM)” heading, we removed the “The Hypothesised Measurement Model” sub-heading. 2) Under the “Hypothesis Testing” heading, we deleted “Motivational Factors (TPB)” AND “Environmental factors LFM” sub-headings. |
| 5 | Overall, more technical details about the SEM can be provided. | Considering your valuable comment, we have added few details about SEM in the revised manuscript, highlighted with green color. |
| 6 | The reference to figures 2 and 3 should be provided in line 393. | We have referenced figures 2 & 3 and highlighted them with green color. We feel great thanks for your point out. |
| 7 | The dataset must be publicly available within either the authors website or a public data repository. | According to your comments and PLOS ONE’s requirements, we have uploaded the data underlying the findings as a Supporting Information file. |
| 8 | Perhaps 83 references are too much. I think that the authors can reduce the references only to the necessary ones. | Thanks for the suggestion, we have reduced the number of references as possible, and we feel that the current references are all important for study. |
| **REVIEWER TWO** | | |
| 1 | The “Methodology” paragraph should be longer and more accurate, highlighting better what has been achieved in the paper and what are the goals that could be achieved in the future. This in order to simplify the successful full understanding of the text by reminding the reader of the logical journey that has been taken while reading the paper | Thanks for your valuable comment. In fact, that paragraph was redundant under the methodology section. Thus, we have restructured the paragraph and shifted it the end of the theoretical part (line 254-257), highlighted with green, reflecting more logical order. Now the methodology section reflects the main components. |
| 2 | There is no clear and ordered reference to the existing literature and methodologies currently in use or to the problems that prevent their full interpretation. I would have given a more schematic and complete visually ordered view to the literature to give a greater basis of credibility to the topic discussed. | Thanks for your comment. If literature review section is referred to here, we believe that it has been comprehensively covered and logically ordered and centered around the main themes the study identified. This no action was taken. |
| 3 | Finally the English language should be checked by a native speaker. | Thank you. The revised manuscript has been cheked by a professional Englsh language editor. |

Kind regards,

Authors
